# Intraoperative Hyperkalemia Due to Surgical Manipulation of a Thymoma

**DOI:** 10.7759/cureus.13758

**Published:** 2021-03-08

**Authors:** John W Mallett, Dustin L Hegland, Joseph C Goldstein

**Affiliations:** 1 Anesthesiology, University of Florida, Gainesville, USA

**Keywords:** hyperkalemia, thymoma, anterior mediastinal mass, tumor lysis syndrome

## Abstract

Episodic hyperkalemia has not been described during resection of a primary thymoma tumor. We present a case of significant intraoperative hyperkalemia during a technically challenging resection of a type B-1 thymoma. The hyperkalemia, presumed to be secondary to considerable tumor manipulation, was successfully controlled with calcium, bicarbonate, and insulin with dextrose. Although strict criteria for tumor lysis syndrome were not met, this possibility was included in the differential diagnosis. This case highlights the importance of close intraoperative electrolyte monitoring and prompt treatment of hyperkalemia during challenging thymoma resection.

## Introduction

Thymomas represent the most common primary anterior mediastinal mass, with an incidence of roughly 1.5 cases per million [1,2]. These rare tumors are typically found in adults, and 30% to 50% of primary thymomas produce myasthenia gravis symptoms [1]. Surgical resection is the standard treatment as thymomas generally do not metastasize, and five-year survival rates often exceed 70% [1-3]. Unilateral phrenic nerve sacrifice, lung lobectomy or resection, pleurectomy, and superior vena cava resection, repair, or prosthetic replacement are well-described surgical tradeoffs associated with complete removal of thymomas, which is commonly performed en bloc with surrounding structures infiltrated by the tumor [3].

Intraoperative hyperkalemia has largely been attributed to lymphomas, abdominal and pelvic tumors, or tumors found in the pediatric population [4-8]. Type B-1 thymomas are predominantly immature T-cells with variable expression of CD-3, CD-4, and CD-8 separated into large lobules and encapsulated by thick, fibrous tissue [9]. Although postoperative tumor lysis syndrome (TLS) after thymoma biopsy has been reported, it has not presented intraoperatively or been associated with significant hyperkalemia [10]. We present a case of intraoperative hyperkalemia during resection of a type B-1 thymoma presumed to be caused by extensive tumor manipulation and highlight the successful treatment using standard therapies, including calcium, bicarbonate, and insulin with dextrose. This case demonstrates the importance of frequent electrolyte assessment and rapid management of hyperkalemia during difficult thymoma resection.

This manuscript was prepared in compliance with the Health Insurance Portability and Accountability Act (HIPAA) of 1996 privacy regulations and adheres to applicable Enhancing the Quality and Transparency of Health Research guidelines (CARES [for CAse REportS] checklist). No adverse outcomes were reported to any manufacturer or governmental regulatory agency. Written patient consent and HIPAA authorization were obtained using a standardized Veteran’s Administration consent form.

## Case presentation

**Figure 1:**
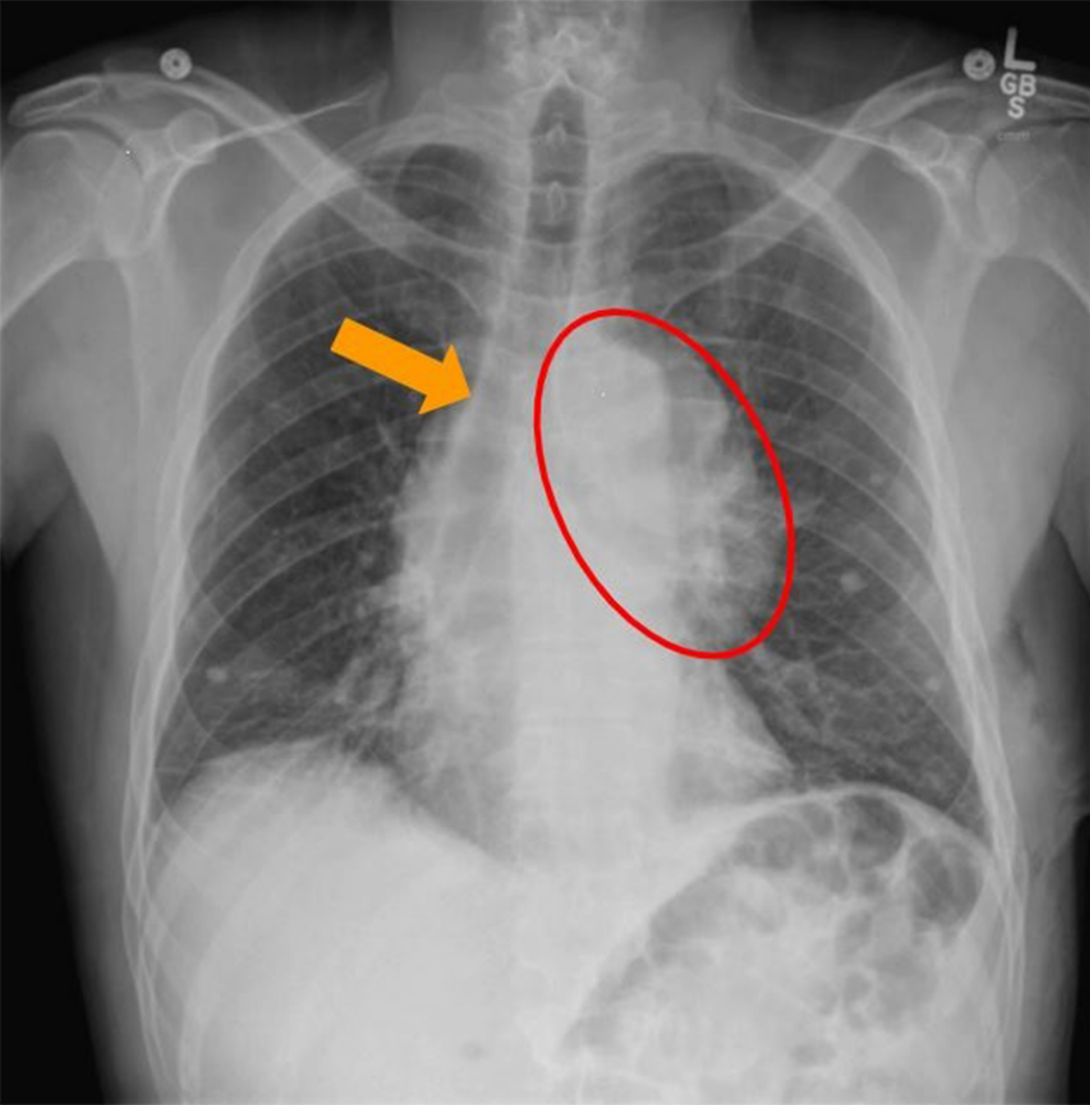
Preoperative chest X-ray. The rightward tracheal shift (orange) due to compression from the left-sided thymoma (red) is shown.

**Figure 2:**
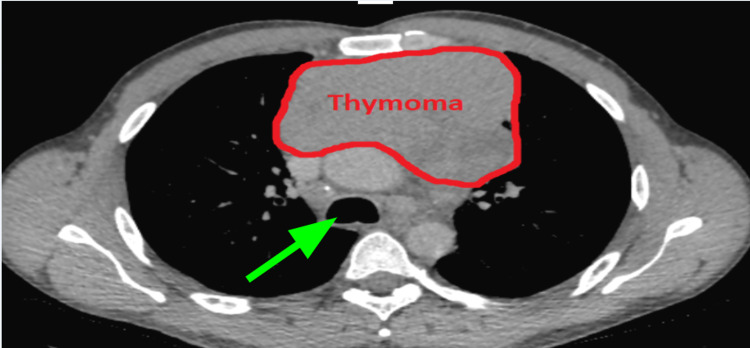
Axial view of 13.1 × 9.4 × 4.5-cm thymoma on computed tomography scan. Rightward deviation of the airway (green arrow) can be noted anterior to the spinal column.

A 185-cm, 75-kg, 46-year-old man with an American Society of Anesthesiologists physical status 3 and a 15 pack-year history of smoking as well as postoperative nausea and vomiting, cannabis use, and gastroesophageal reflux presented for median sternotomy with open resection of a left-sided, biopsy-proven 13.1 × 9.4 × 4.5-cm type B-1 thymoma with distal tracheal deviation (Figures 1, 2). The patient had initially presented with unrelated symptoms that led to a chest X-ray. This incidentally revealed the mediastinal mass, which was subsequently further evaluated. The patient had no family history of muscular dystrophies or electrolyte abnormalities. Although the original frozen section was read as lymphoma, final cytology confirmed thymic origin.

On the morning of the surgery, the patient denied dyspnea, orthopnea, double vision, difficulties chewing, speaking, or swallowing, or peripheral muscle weakness. Airway examination revealed a Mallampati IV classification with appropriate mouth opening and prognathism without restriction of neck movement. No stridor was appreciated in the sitting or supine position.

After discussion with the procedural team about vessels at risk for surgical sacrifice, a 20-G left radial arterial catheter was placed preoperatively using local anesthesia. Preoperative arterial blood gas (ABG) and other laboratory studies showed no significant abnormalities, preserved renal function with normal estimated glomerular filtration rate, a serum creatinine of 1.0 mg/dL, a serum blood urea nitrogen of 14 mg/dL, and a serum potassium of 4.5 mmol/L.

Anxiolysis was achieved using midazolam, and the patient was induced with intravenous ketamine combined with sevoflurane via a nasal continuous positive airway pressure device (SuperNO2VA^TM^; Vyaire Medical, Mettawa, IL, USA), ensuring preservation of spontaneous ventilation. A 20-mg bolus of intravenous propofol was administered to facilitate placement of a size 4 igelⓇ supraglottic airway (Intersurgical Ltd., Wokingham, Berkshire, UK). Bronchoscopy was performed through the supraglottic airway to obtain visualization of the left main bronchus and secondary carina. Propofol was incrementally titrated until spontaneous ventilation was temporarily halted and capacity to provide adequate positive pressure ventilation was ensured. Reassuring bronchoscopy findings and ventilation allowed titration of rocuronium and uneventful placement of a left-sided 37-French, double-lumen endotracheal tube via direct laryngoscopy. Appropriate positioning was confirmed under bronchoscopic visualization.

**Figure 3:**
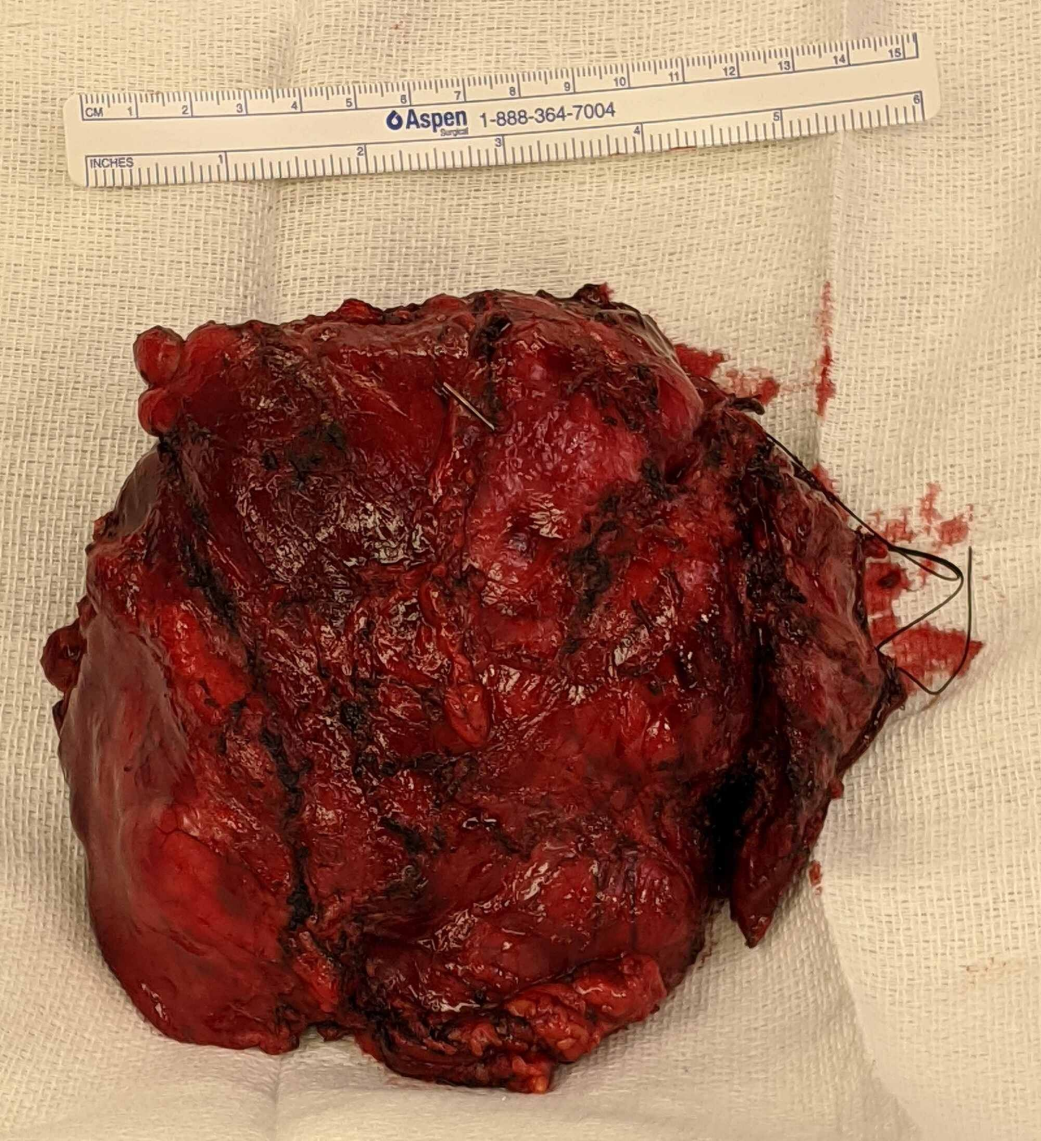
Gross specimen immediately post-excision. A 15-cm measuring tape is located at the top of the picture for reference.

The surgical resection was technically challenging given local tumor invasion into the left lung parenchyma as well as bilateral mediastinal parietal pleura. The resection required significant tumor manipulation, and the left phrenic nerve could not be spared by the cardiothoracic surgeons (Figure 3). To help improve surgical conditions, intermittent alternating left and right lung isolation was required. The patient tolerated this well without significant desaturation. Hypercarbia levels were maintained within acceptable ranges anticipated for one-lung ventilation, especially compared to thoracoscopically assisted cases with carbon dioxide insufflation.

After considerable tumor manipulation requiring intermittent breath holds, an intraoperative ABG was obtained to assess oxygenation and ventilation status, revealing a potassium of 6.5 mmol/L. To confirm this value, another ABG drawn by a different physician showed a potassium of 6.2 mmol/L. In an effort to remove further confounding factors, a different anesthesia technician ran a third confirmatory ABG that returned a potassium of 6.1 mmol/L, a pH of 7.27, partial pressure of carbon dioxide of 56 mmHg, hemoglobin of 12.6 g/dL, bicarbonate of 24.7 mmol/L, a base deficit of 2.4 mmol/L, a sodium of 133 mmol/L, a chloride of 107 mmol/L, ionized calcium of 1.09 mmol/L, and a glucose of 133 mg/dL (Table 1). T-waves were not peaked, and there were no other notable abnormalities on electrocardiogram.

**Table 1:** Arterial blood gas values.

	08:10 hrs (preoperative)	13:28 hrs	13:38 hrs	13:52 hrs	15:03 hrs (surgery completion)	17:07 hrs (postoperative)
pH	7.432	7.267	7.276	7.282	7.294	7.314
pCO^2^ (mmHg)	35.5	55.9	53.8	53.1	51.3	50.6
pO^2^ (mmHg)	73.8	99.3	99.8	90.2	159	78.8
Na (mmol/L)	136	133	133	133	133	---
K (mmol/L)	4.5	6.5	6.2	6.1	5.2	4.8
Cl (mmol/L)	108	107	109	107	107	---
HCO^3^ (mmol/L)	23.4	24.7	24.3	24.3	24.1	25.1
Glucose (mg/dL)	103	133	125	119	121	---
Ionized calcium (mmol/L)	1.15	1.09	1.06	1.06	1.39	1.27
Hemoglobin (g/dL)	12.7	12.6	12.3	12.4	11.2	12.2
Base deficit (mmol/L)	0.2	2.5	2.6	2.5	2.2	1.2

The patient was given 50 mEq of sodium bicarbonate and 6 g of calcium gluconate over 45 min. A five-unit bolus of insulin was administered intravenously with 12.5 g of dextrose, and an insulin infusion was started at 2 units/hour and titrated up to 5 units/hour. Given frequent one-lung ventilation and intermittent respiratory cessation, β-agonist therapy and hyperventilation were deferred. Diuretic therapy was avoided given implementation of other therapies, adequate urine production, and a lack of electrocardiographic changes or other symptoms. The patient’s potassium level improved to 5.2 mmol/L by the end of the procedure without evidence of hypoglycemia, and he produced 800 mL of urine. The patient’s temperature was 36.4°C at the beginning, 36.1°C during, and 37.1°C at the end of the surgery. After successful conclusion of the procedure, the patient’s respiratory mechanics were evaluated using negative inspiratory force and rapid shallow breathing index, and he was extubated uneventfully and transferred to the intensive care unit. The patient did not experience any further hyperkalemia during his hospital stay and was discharged on postoperative day 3. At follow-up, the patient expressed appreciation for the care he received.

## Discussion

Although postoperative TLS has occurred after thymoma biopsy, prior evidence of isolated intraoperative hyperkalemia attributable to tumor manipulation is lacking [10]. A case report by Trobaugh-Lotrario et al. mentioned postoperative TLS with “marked changes” in chemistries; however, potassium levels reached a maximum of 5.1 mmol/L, and yet their patient ultimately required dialysis for renal failure [10]. Similar to their case report, the preliminary frozen section in our patient was also read as lymphoma, and only the final pathological report identified the tumor as a primary thymoma [10]. This alludes to the diagnostic challenges that a primary thymoma can present; lung metastases, lymphoma, goiter, and thymic carcinomas can also present as anterior mediastinal masses [1,2]. Type B-1 thymomas such as the one seen in our patient are considered lymphocyte-rich thymomas and have a thick capsule [10].

TLS is rarely associated with thymic tumors; only a few case reports detail this phenomenon after chemotherapy induction [10]. Criteria for TLS (at least two of the following laboratory changes in proximity to cytotoxic therapy: hyperuricemia, hyperphosphatemia, hyperkalemia, or hypocalcemia) or clinical diagnosis of TLS (cardiac, renal, or neurological sequelae stemming from metabolic disturbances in proximity to cytotoxic therapy) were never met in this case [11]. However, due to the absence of clinical findings, uric acid levels were not drawn perioperatively, preventing complete exclusion of this diagnosis. While hyperkalemia required intraoperative treatment, no other electrolyte disturbances required intervention.

Pseudohyperkalemia, or hyperkalemia stemming from hemolysis of red blood cells, is most often caused by aggressive phlebotomy or sample handling [12]. This was ruled out by having multiple physicians carefully draw the ABG sample and multiple technicians run the samples. While excessive potassium intake or administration may also affect potassium levels, only balanced crystalloid solution was administered without supplementation of potassium to our patient, there was no evidence of intravascular hemolysis, and the patient received no intraoperative blood products [12].

Prerenal and intrinsic renal damage typically lead to prolonged hyperkalemic episodes [12]. No significant postoperative hyperkalemia occurred, and the patient maintained appropriate urine output during the procedure. There were no instances of profound hypotension intraoperatively that may have contributed to renal hypoperfusion injury. Follow-up laboratory studies in the intensive care unit did not indicate renal dysfunction.

Cellular redistribution can cause hyperkalemia through numerous underlying mechanisms [12]. In this case, ABG showed respiratory acidosis, which is a potential cause of hyperkalemia. However, this is still less likely to have resulted in a notable potassium shift as significant hyperkalemia has not been commonly demonstrated during one-lung ventilation, and new evidence indicates that respiratory acidosis likely has a minimal effect on serum potassium levels [13,14]. Beta-blockers can reduce cellular uptake of potassium ions; however, no perioperative beta-blockade was administered [12]. Although insulin deficiency can lead to hyperkalemia, the preoperative ABG showed a glucose of 108 mg/dL, reducing the probability that this was the cause of the hyperkalemia. Finally, hypovolemia and hyperkalemic periodic paralysis can lead to hyperkalemia, but with successful extubation at the conclusion of the procedure and adequate fluid resuscitation intraoperatively, these are possible, but less likely [12]. Cellular injury appears to be the most likely etiology, with tumor manipulation being the most probable underlying reason.

## Conclusions

In conclusion, we present an unusual case of isolated hyperkalemia without electrocardiographic findings presumed secondary to tumor manipulation during a challenging resection of a type B-1 thymoma. The patient’s hyperkalemia was managed using calcium gluconate, sodium bicarbonate, and a bolus of insulin and dextrose with subsequent insulin infusion. Given no evidence of recurrence of hyperkalemia postoperatively, this phenomenon appears limited in duration, requiring only acute temporization to prevent malignant arrhythmias with subsequent spontaneous electrolyte improvement in patients with preserved renal function. Electrolyte values should be monitored closely during the resection of thymomas given the potential for hyperkalemia secondary to tumor manipulation, which may potentially herald preventable cardiac arrhythmias.
